# Supplementary figures and images for: TRPM8 modulates temperature regulation in a sex-dependent manner without affecting cold-induced bone loss
Source: PLoS One. 2021 Jun 4;16(6):e0231060. doi: 10.1371/journal.pone.0231060 (PMC8177490; doi:10.1371/journal.pone.0231060)

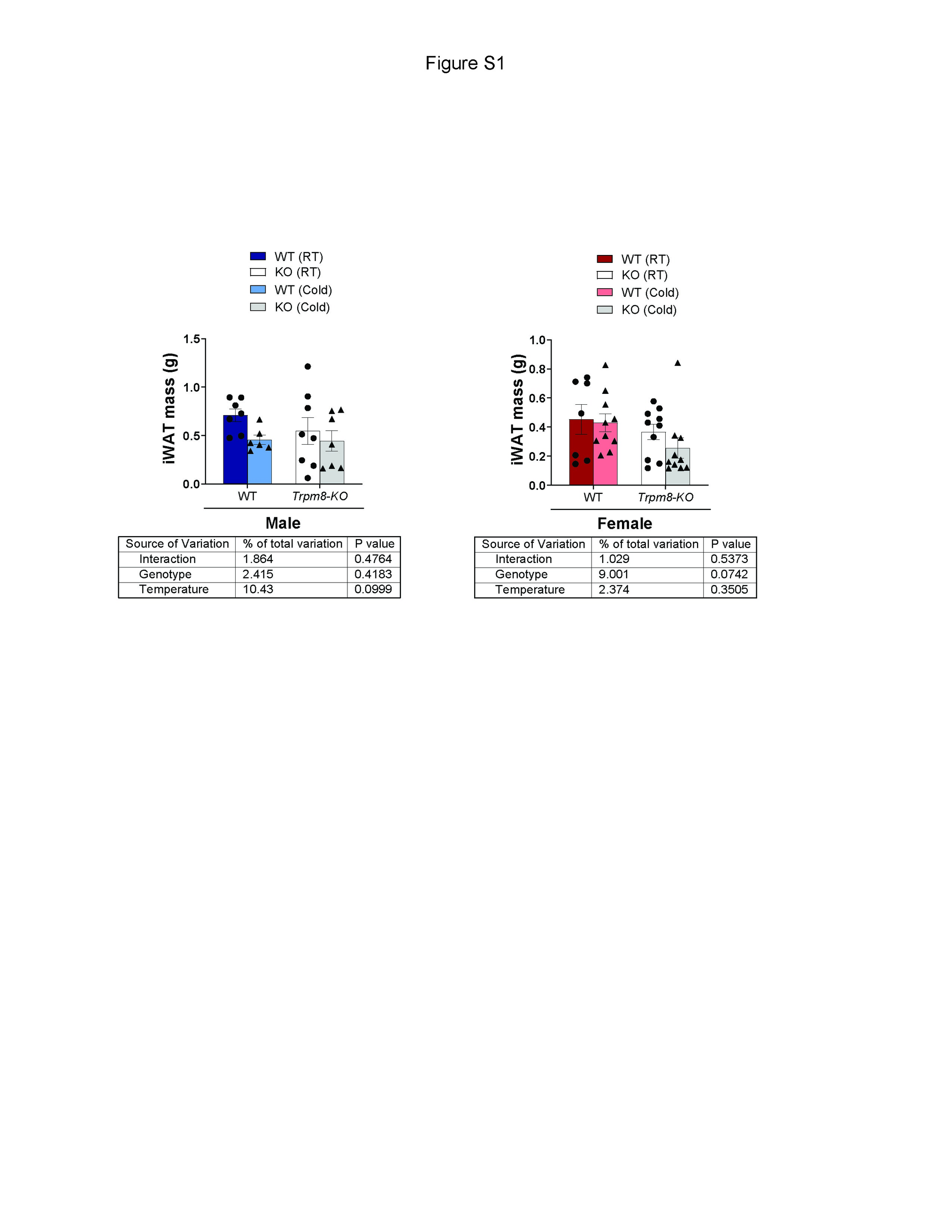

Supplement: S1 Fig — N = 6–10. (TIF) [file pone.0231060.s001.tif]
