# Supplementary material for: TRPM8 modulates temperature regulation in a sex-dependent manner without affecting cold-induced bone loss
Source: PLoS One. 2021 Jun 4;16(6):e0231060. doi: 10.1371/journal.pone.0231060 (PMC8177490; doi:10.1371/journal.pone.0231060)
Supplement: S1 Table — (DOCX) [file pone.0231060.s002.docx]

**S1 Table.** **qPCR primer information.**

| **Gene** | **Source** | **Sequence** | **Reference** |
| --- | --- | --- | --- |
| *Acadl* | Primer Design | For: 5'-GGA ATG AAA GCT CAG GAC ACA -3' | N/A |
|  |  | Rev: 5'-AGC CTT TAT TCT CTT CTC CAA GT -3' |  |
| *Cidea* | IDT | For: 5'-TGC TCT TCT GTA TCG CCC AGT -3' | Seale et al. (2008) |
|  |  | Rev: 5'-GCC GTG TTA AGG AAT CTG CTG -3' |  |
| *Dio2* | IDT | For: 5'-CAG TGT GGT GCA CGT CTC CAA TC -3' | Cooper et al. (2008) |
|  |  | Rev: 5'-TGA ACC AAA GTT GAC CAC CAG -3' |  |
| *Pdk4* | Primer Design | For: 5'-AAA GTG GGT CTG TGG CAT TG -3' | N/A |
|  |  | Rev: 5'-AAG GTT TGT ACT CGT GTT TGT G -3' |  |
| *Pparc1α* | IDT | For: 5'- TGA TGT GAA TGA CTT GGA TAC AGA CA -3' | Cooper et al. (2008) |
|  |  | Rev: 5'- GCT CAT TGT TGT ACT GGT TGG ATA TG -3' |  |
| *Pparγ2* | IDT | For: 5'-GCA TGG TGC CTT CGC TGA -3' | Cooper et al. (2008) |
|  |  | Rev: 5'-TGG CAT CTC TGT GTC AAC CAT G -3' |  |
| *Prdm16* | IDT | For: 5'-CAG CAC GGT GAA GCC ATT C -3' | Cooper et al. (2008) |
|  |  | Rev: 5'-GCG TGC ATC CGC TTG TG -3' |  |
| *Tbp1* | IDT | For: 5'-GAA GCT GCG GTA CAA TTC CAG -3' | Sanchez-Gurmaches and Guertin (2014) |
|  |  | Rev: 5'-CCC CTT GTA CCC TTC ACC AAT -3' |  |
| *Ucp1* | IDT | For: 5'-ACT GCC ACA CCT CCA GTC ATT-3' | Cooper et al. (2008) |
|  |  | Rev: 5'- CTT TGC CTC ACT CAG GAT TGG -3' |  |

N/A: not applicable.

**Supporting References**

Cooper, M.P., Uldry, M., Kajimura, S., Arany, Z., Spiegelman, B.M., 2008. Modulation of PGC-1 coactivator pathways in brown fat differentiation through LRP130. J. Biol. Chem. 283, 31960–31967. https://doi.org/10.1074/jbc.M805431200

Sanchez-Gurmaches ; Guertin, D., 2014. Adipocytes arise from multiple lineages that are heterogeneously and dynamically distributed Access. Nat Commun. 5, 4099. https://doi.org/10.1038/ncomms5099

Seale et al., 2008. PRDM16 Controls a Brown Fat/Skeletal Muscle Switch. Bone 23, 1–7. https://doi.org/10.1038/nature07182
